# Resistance to an eriophyid mite in an interspecific hybrid pedigree of *Populus*

**DOI:** 10.1371/journal.pone.0207839

**Published:** 2018-11-26

**Authors:** George Newcombe, Wellington Muchero, Posy E. Busby

**Affiliations:** 1 College of Natural Resources, University of Idaho, Moscow, Idaho, United States of America; 2 Biosciences Division, Oak Ridge National Laboratory, Oak Ridge, Tennessee, United States of America; 3 Botany and Plant Pathology Department, Cordley Hall, Oregon State University, Corvallis, Oregon, United States of America; Austrian Federal Research Centre for Forests BFW, AUSTRIA

## Abstract

*Schizoempodium mesophyllincola* is an eriophyid mite that feeds in leaves of *Populus trichocarpa* in the central part of this cottonwood tree’s range (i.e., coastal British Columbia, Washington and Oregon) in the Pacific Northwest (PNW) of North America, and on some interspecific hybrids planted in short-rotation, intensive forestry in the region. The mite, a leaf vagrant, sucks the contents of spongy mesophyll cells, causing leaf discoloration, or “bronzing.” Here, we investigate the inheritance pattern of resistance to leaf bronzing using a three-generation *Populus trichocarpa* x *P*. *deltoides* hybrid pedigree. We found that resistance to the mite is an exaptation in that its source in two related F_2_ families of the TxD hybrid pedigree was the non-native host, *P*. *deltoides*. Two grandparental genotypes of the latter, ‘ILL-5’ and ‘ILL-129’, were completely free of the bronzing symptom and that phenotype was inherited in a Mendelian manner in the F_1_ and F_2_. Resistance to *S*. *mesophyllincola* is similar to resistance to many other regional pathogens of *P*. *trichocarpa* (e.g., *Melampsora occidentalis*, *Venturia inopina*, *Sphaerulina populicola*, and *Taphrina* sp.) in that it is inherited from the non-native grandparent (e.g., *P*. *deltoides*, *P*. *nigra*, or *P*. *maximowiczii*) in three-generation, hybrid pedigrees. In addition to finding evidence for Mendelian inheritance, we found two QTLs with LOD scores 5.03 and 3.12 mapped on linkage groups (LG) III and I, and they explained 6.7 and 4.2% of the phenotypic variance, respectively. The LG I QTL is close to, or synonymous with, one for resistance to sap-feeding arthropods and leaf developmental traits as expressed in a British study utilizing the same pedigree.

## Introduction

The genetic basis of resistance to pathogens of *Populus trichocarpa* has frequently been inferred from studies of its interspecific hybrid pedigrees deployed in the native range of this cottonwood species in the Pacific Northwest of North America [1]. Resistance to fungal diseases is often inherited from the non-native species (e.g., *P*. *deltoides* or *P*. *maximowiczii*) in such pedigrees [2–5] and is therefore an example of exaptation in that such resistance is co-opted for its current role [5]. Genetic resistance to an insect was an exception [6], and other complexities were discovered in the past. For example, in the case of hybridization between the native rust in the range of *P*. *trichocarpa*, and the rust introduced to the region with *P*. *deltoides* [7], genes for resistance were identified in *P*. *trichocarpa* but also in *P*. *deltoides* [8,9].

Even though major genes for resistance are typically not inherited from *P*. *trichocarpa* in response to its own regional pathogens, other mechanisms of resistance are possible. For example, native cottonwood leaves also host endophytes that can antagonize pathogens [10,11]. Cottonwood leaves can even host hyper-parasites, or mycoparasites (e.g., *Eudarluca caricis*–[12]; *Hydropisphaera fungicola–* [13]) so resistance to any particular pathogen can involve complex interactions among microorganisms. This study was motivated by the desire to further develop our knowledge of that community, and in particular, to investigate patterns of resistance to an endophytic, eriophyid mite.

**Fig 1:**
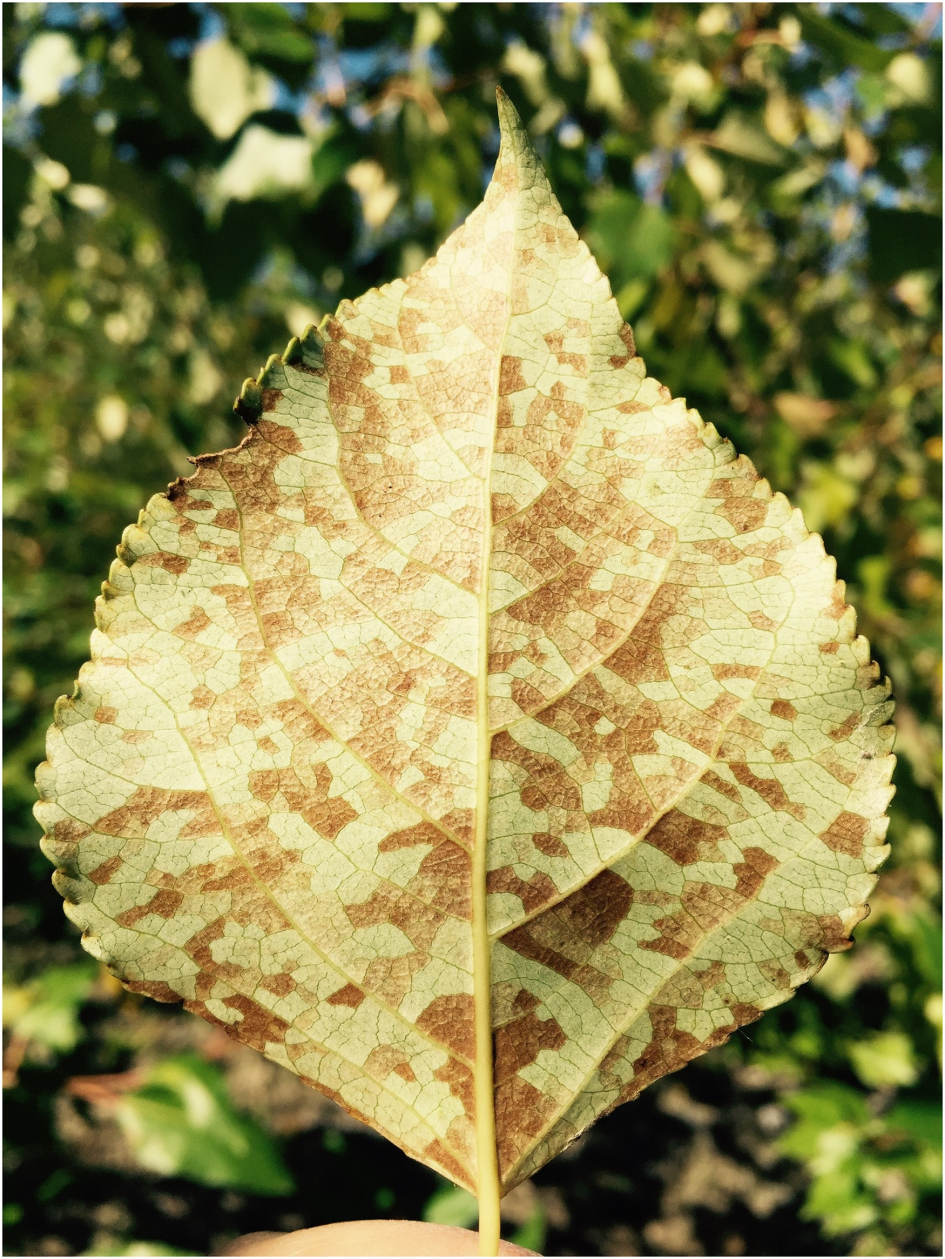
Leaf bronzing on *Populus trichocarpa* caused by *Schizoempodium mesophyllincola*.

*Schizoempodium mesophyllincola* Oldfield, Hunt, and Gispert was described as a new genus and species of host-specific eriophyid mite almost twenty years ago [14]. This eriophyid mite is endemic to the maritime Pacific Northwest west of the Cascades. It not only causes leaf bronzing in its native host, *Populus trichocarpa*; it also affects advanced-generation hybrids of *Populus trichocarpa* x *P*. *deltoides* (TxD), although not TxD F_1_s [15]. Bronzing can be severe on some clones, yet others are completely resistant. The mite feeds on spongy mesophyll cells by piercing them and then sucking out the cytoplasm. It accesses mesophyll cells via stomata of the abaxial surface of leaves, and it is only this surface that becomes bronzed. Bronzing can become very conspicuous in the most susceptible hybrids by summer (Fig 1).

Our objectives in this study were to evaluate segregation of resistance to *S*. *mesophyllincola* in two *Populus trichocarpa* x *Populus deltoides* hybrid pedigrees. For families 331 and 355 we combined mite bronzing phenotypic data with a genetic linkage map in the F_2_ family 331 to map quantitative trait loci (QTLs) underlying resistance to *S*. *mesophyllincola*.

## Materials and methods

### Mapping pedigree

We used a pedigree, common-garden study to evaluate genetic resistance to *S*. *mesophyllincola*. Mite damage (referred to as “bronzing” due to the physical appearance of damage, Fig 1) was scored on approximately 600 tree genotypes in two successive years (1994, 1995) in September in a three-generation *Populus trichocarpa* x *Populus deltoides* hybrid pedigree near Clatskanie, OR (for more details see [16]). The planting included a total of six trees per genotype: 376 genotypes of F_2_ family 331, its F_1_ parents, and its *P*. *trichocarpa* (93-968) and *P*. *deltoides* grandparents (ILL-129), and 230 genotypes of F_2_ family 355, its F_1_ parents, and its *P*. *trichocarpa* (93-968) and *P*. *deltoides* grandparents (ILL-5). The two families shared a common *P*. *trichocarpa* grandmother, but had different *P*. *deltoides* grandfathers.

### Phenotypic data

We used leaf bronzing to estimate the severity of mite damage. Leaf bronzing is caused by mite feeding within the spongy mesophyll contiguous with the lower, abaxial surface of leaves [15]. The proportion of leaf area bronzed correlates with the number of mites feeding within a leaf [15]. We scored bronzing on each tree using the Schreiner scale [2]. First, bronzing was estimated as light (score = 1), medium (score = 5) or heavy (score = 25) on the most severely damaged leaves. Next, the percentage of the leaf bronzed was estimated at <25 (score = 1), 26–50 (score = 2), 51–75 (score = 3), or >75 (score = 4). Finally, the two scores were multiplied to give the composite rating from 0 (highly resistant) to 100 (highly susceptible).

To evaluate whether a single, major gene confers resistance to the mite, with the resistant allele dominant (i.e., Mendelian inheritance), we calculated the proportion of resistant and susceptible genotypes in the two F_2_ families. We used a cut-off value of clone means of ≤5 for the resistant class and >5 for the susceptible class. Individuals in the resistant class exhibited no symptoms other than such light ‘bronzing’ of some leaves that it would be difficult to unequivocally attribute the symptoms to the mite. We used Chi-square tests to evaluate whether the proportion of resistant and susceptible genotypes in the two F_2_ families are consistent with Mendelian inheritance. Normality of the phenotypic data in family 331 was assessed using the Anderson-Darling test before QTL mapping was performed.

### QTL analysis

Genotyping of the family 331 pedigree, map creation and the QTL mapping approach were previously described [17,18]. Briefly, a genome-anchored map comprising 841 AFLP, RAPD, RFLP and SSR markers was used to localize QTL intervals. The Multiple-QTL Model (MQM) package of MapQTL 6.0 software [19] with automatic cofactor selection was used to map putative QTL intervals on the family 331 genetic map. The regression algorithm, LOD test statistic and fit dominance for F_2_ were used to identify QTL intervals with a mapping step size of 1.0, maximum number of neighboring markers of 5, maximum number of iterations of 200 and a 1.0E-08 functional tolerance value. Since the phenotypic data exhibited non-normality from the Anderson-Darling test above, the non-parametric Kruskal-Wallis (KW) analysis with a significance threshold of 0.005 was used to confirm MQM results. Permutations were conducted separately for each linkage group and LOD significance thresholds were calculated using 1000 permutations at the 0.05 significance level.

## Results

### Mendelian resistance to the mite

**Fig 2:**
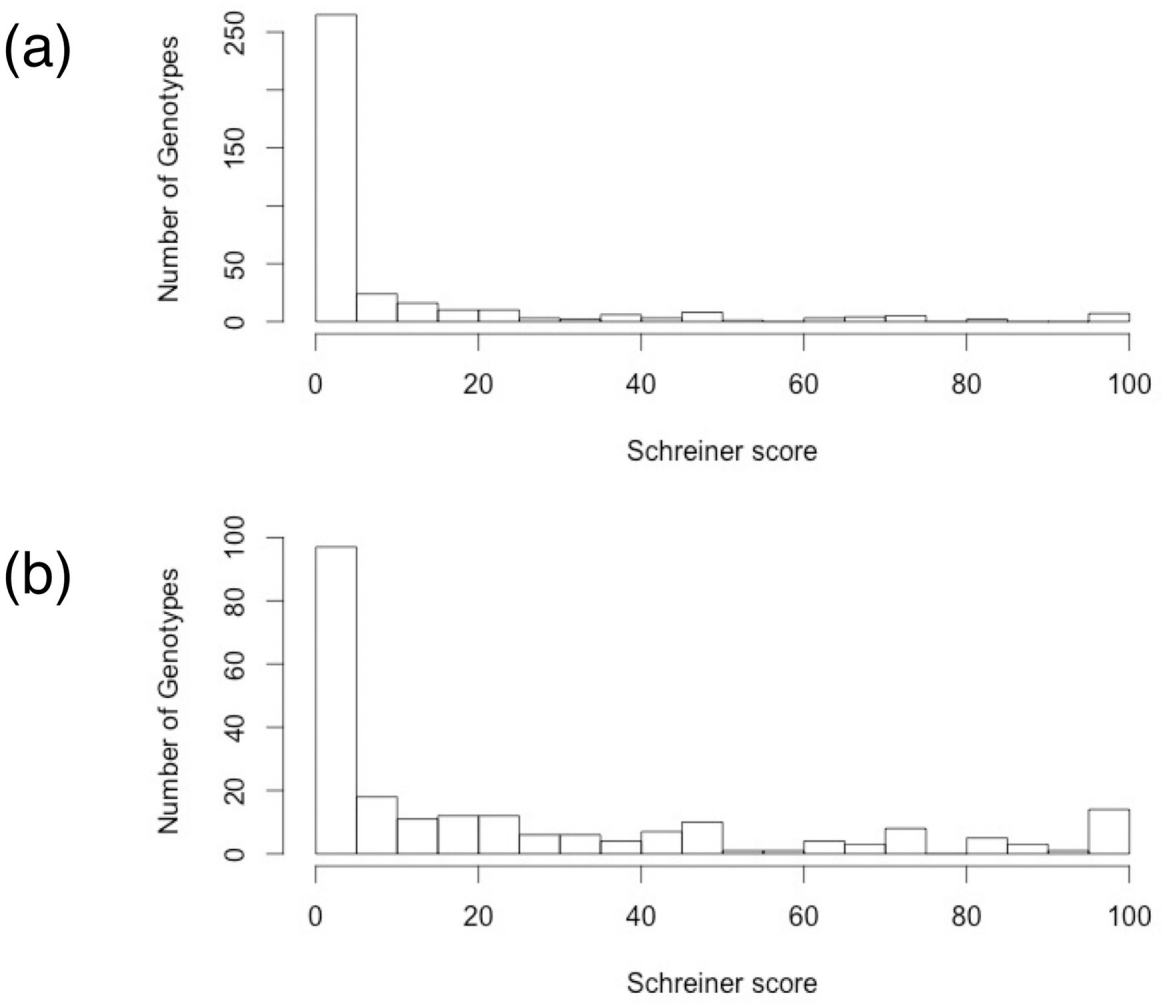
Histogram of Schreiner scores for bronzing in F_2_ families 331 (a) and 355 (b).

**Fig 3:**
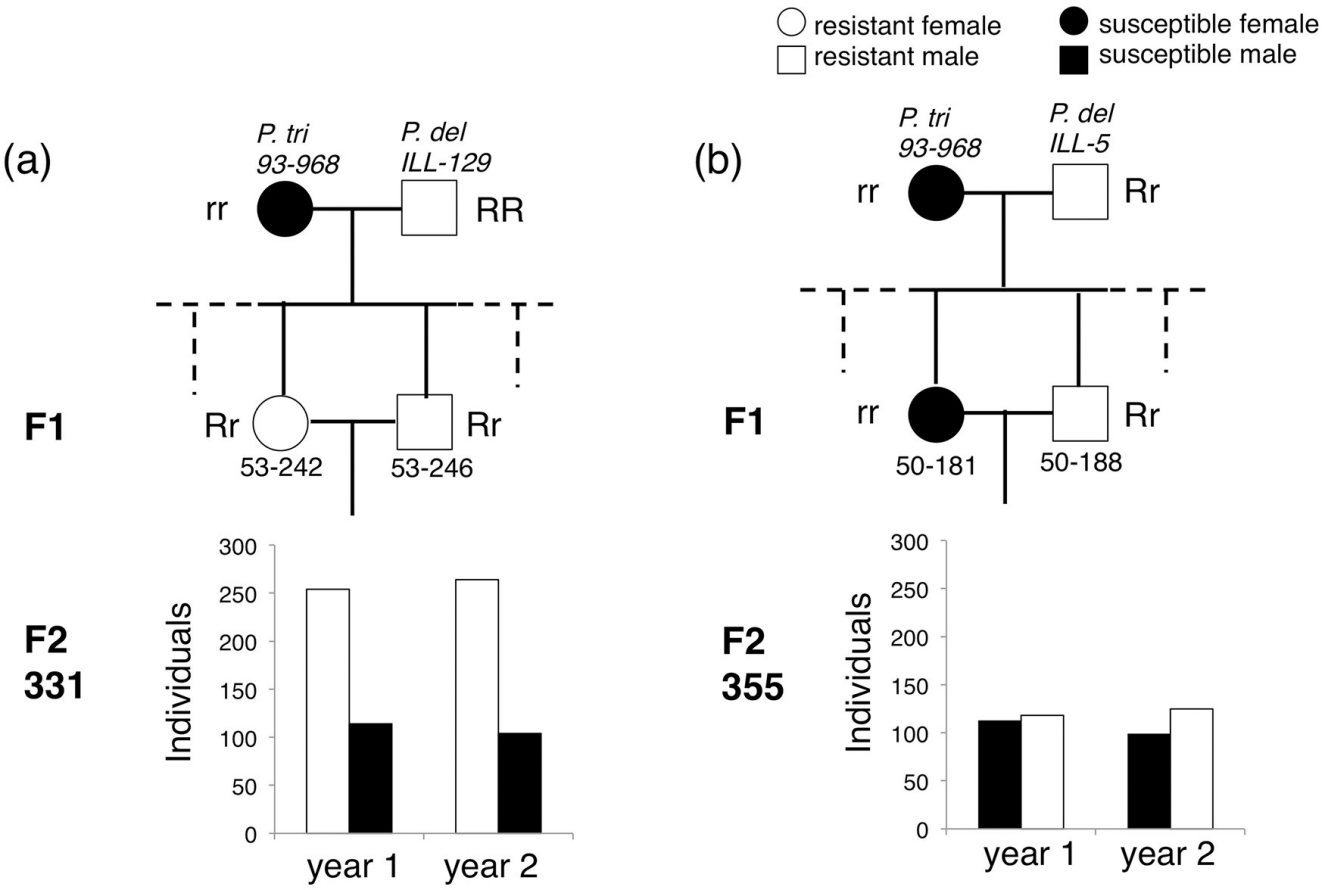
Three-generation pedigree for two F_2_ families: 331 (a) and 355 (b) depicting resistant (damage≤5) and susceptible (damage >5) phenotypes. A Mendelian pattern of resistance to bronzing is evident with 1:1 and 3:1 ratios of resistant:susceptible in the two related F_2_ families.

The distribution of Schreiner scores observed in the three-generation pedigree is shown in Fig 2. With bronzing scores of either ‘resistant’ (≤5) or ‘susceptible’ (>5), we observed a Mendelian pattern of resistance: 1:1 and 3:1 ratios of resistant:susceptible phenotypes in the two related F_2_ families (Fig 3). The dominant allele for bronzing resistance was thus inherited from two different male *P*. *deltoides* clones from Illinois. The F_1_ parents of the F_2_ family 331 in which resistance segregates 3:1 are both resistant and heterozygous (Fig 3a). The F_1_ parents of the F_2_ family 355 in which resistance segregates 1:1 are phenotypically resistant and susceptible to *S*. *mesophyllincola*, and genotypically heterozygous and homozygous recessive, respectively (Fig 3b).

#### Family 355

In both study years, the *P*. *trichocarpa* clone 93–968 was susceptible, whereas the two *P*. *deltoides* clones were completely resistant (i.e., Schreiner scores of 0) (Table 1; Fig 3). One F_1_ parent, 50–188, was resistant to bronzing in both study years; however, its sibling 50–181, was susceptible (Table 1; Fig 3). In the first year, there were 113 clones in F_2_ family 355 with bronzing means ≤5, and 118 with bronzing means >5. These genotypes were considered resistant and susceptible, respectively. A 1:1 segregation for resistance:susceptibility would be expected for a single resistance gene if the resistant parent, 50–188, is heterozygous for this dominant gene for resistance, and the susceptible parent, 50–181, is homozygous recessive. The 113:118 observed ratio of resistant to susceptible siblings did not differ from the expected 1:1 ratio (Pearson Chi-square = 0.054, P = 0.82; Yates corrected Chi-square = 0.019, P = 0.889). In the second year, the segregation again did not differ significantly from the expected 1:1 ratio (99 resistant:125 susceptible; Pearson Chi-square = 1.514, P = 0.219; Yates corrected Chi-square = 1.290, P = 0.256) (Fig 3).

**Table 1:** Susceptibility (S: mean severity >5) or resistance (R: mean severity ≤5) to leaf bronzing caused by *Schizoempodium mesophyllincola*, of the F_1_ parents and grandparents of the two related F_2_ families.

	Mean bronzing severity in year 1	Mean bronzing severity in year 2
*P*. *trichocarpa*, clone 93–968 (parent of F_1_ family 50 and 53, grandparent of F_2_ family 355 and 331)	21.7 (S)	33.8 (S)
*P*. *deltoides*, clone ILL-5 (parent of F_1_ family 50, grandparent of F_2_ family 355)	0 (R)	0 (R)
*P*. *deltoides*, clone ILL-129 (parent of F_1_ family 53, grandparent of F_2_ family 331)	0 (R)	0 (R)
F_1_ clone 50–181	22.5 (S)	31.3 (S)
F_1_ clone 50–188	3.5 (R)	3.8 (R)
F_1_ clone 53–242	3.8 (R)	0.5 (R)
F_1_ clone 53–246	4.8 (R)	4.3 (R)

#### Family 331

Both F_1_ parents, 53–242 and 53–246, were resistant to bronzing in both study years (Table 1; Fig 3). In the first year, 254 clones in F_2_ family 331 had bronzing means ≤5 and were thus considered resistant; 114 genotypes had bronzing means >5 and were considered susceptible. A 3:1 segregation for resistance:susceptibility would be expected for a single gene for resistance in family 331 if the two F_1_ parents were resistant heterozygotes. The 254:114 observed ratio of resistant to susceptible siblings did not differ from the expected 3:1 ratio (Pearson Chi-square = 3.263, P = 0.071; Yates corrected Chi-square = 2.973, P = 0.085). In the second year, the segregation again did not differ significantly from the expected 3:1 ratio (264 resistant:104 susceptible; Pearson Chi-square = 1.001, P = 0.317; Yates corrected Chi-square = 0.841, P = 0.359; Fig 3).

### QTL analysis

With the full range of 13 Schreiner-scale categories of bronzing severity in family 331 we found two QTLs with LOD scores 5.03 and 3.12 that mapped on LGs III and I (Table 2). They explained 6.7 and 4.2% of the phenotypic variance, respectively (Table 2). The two QTLs were also significant based on the non-parametric Kruskal-Wallis analysis, with a probability of the maximum achievable level of 0.0001 (Table 2). A third, suggestive QTL was identified on LG V between SSR markers WSPM_15 and PMGC_2839 with a LOD score of 2.02 and Kruskal-Wallis significance of 0.0005 (Table 2).

**Table 2:** Results of multiple-interval QTL mapping and Kruskal-Wallis analysis of mite damage.

QTL interval (LG: cM range)	Nearest marker	MQM analysis		Kruskal-Wallis	
		LOD score	%PVE^†^	K-value	p-value
LGIII: 33.642–59.393	ORPM_26	5.03	6.7	17.07	0.0001
LGI: 108.686–118.167	PMGC_2889	3.12	4.2	22.86	0.0001
LGIV:63.447–76.062	WPMS_15	2.02	2.7	12.13	0.0005

^†^%PVE = percent phenotypic variance explained.

## Discussion

Eriophyid mites cause many distinct disorders of trees by feeding on leaves, buds, stems, flowers, and fruit [20]. Our determination of Mendelian inheritance of resistance to *S*. *mesophyllincola* is a first for any arboreal eriophyid mite, to the best of our knowledge. The strong host specificity of *S*. *mesophyllincola* is like that of fungal pathogens [1–5] and other eriophyid mites [14,15], but unlike many poplar-feeding insects [6].

The LG I QTL identified in our study is close to, or synonymous with, one for resistance to sap-feeding arthropods (i.e., aphids) and leaf developmental traits as expressed in a recent study in the UK utilizing the same *Populus* pedigree [21]. A number of possible mechanisms of resistance segregate among F_2_ individuals (i.e., family 331) in the interspecific, three-generation pedigree of *Populus trichocarpa* x *P*. *deltoides*, or TxD hybrids. Family 331 has also been used in many other analyses of resistance in TxD hybrids where the T and D traits contrast strongly. With respect to *S*. *mesophyllincola*, bronzing is compartmentalized in areas delimited by leaf veins, and ‘vein density’ is much greater in the resistant *P*. *deltoides* than in susceptible *P*. *trichocarpa*. Another morphological difference in leaves of *P*. *deltoides* and *P*. *trichocarpa* is the color of the abaxial surface. In *P*. *trichocarpa*, this surface is reflective and appears white, due to large intercellular airspaces. Large intercellular airspaces are lacking in the bronzing-resistant *P*. *deltoides*, and thus this is also a possible mechanism of resistance. A third putative mechanism of resistance is suggested by the large cross-sectional area of mites relative to stomatal apertures [15]; small apertures could prevent mites from entering and exiting. Fourth, because mites need to colonize leaves in spring, the timing of spring flush could be a phenological mechanism of resistance. Thus, of the 14 QTLs identified in the DeWoody study [21], it was the multiple, leaf-trait hotspot that included the QTL for sap-feeding arthropods (i.e., aphids) on leaves in August that seemed most likely to be significant in this study of resistance to *S*. *mesophyllincola*. It is noteworthy that this is in fact one of the three that we identified: on LGI I and mapped in exactly the same interval that was previously identified by DeWoody [21]. In that study, genomic hotspots for resistance to aphids as well as leaf morphological traits (including leaf width, length and area) mapped to two separate hotspots on LGs 1 and X11. In our study, SSR markers delimiting the DeWoody QTLs (i.e., PMGC_2889, PMGC_2885 and PMGC_495), mapped in the same QTL interval on LG1 of the genome-anchored map reported by Muchero [18], suggesting that these are actually the same interval that was split into two LGs in previous maps due to insufficient marker coverage.

It is also interesting that our result differs from that of an earlier paper on resistance in a willow pedigree [22]. In that study resistance to an eriophyid mite, *Aculops tetanothrix*, was not associated in F_2_s with resistance to an aphid. Possibly the difference between the studies might have to do with the ecology of the specific eriophyid mites. *Aculops tetanothrix* causes galls rather than leaf bronzing, and that may represent a significant, feeding-based divergence.

It is important to note that most hybrids in poplar plantations are not susceptible to bronzing for reasons that were made clear by this study. TxD F1s are generally resistant given the mode of inheritance reported here. Interspecific hybrids with sources of non-native resistance other than *P*. *deltoides* (e.g., *P*. *nigra*, N, or *P*. *maximowiczii*, M) also appear to be resistant. Thus, similar results to the ones reported here (i.e., dominant resistance prevailing in F1s but segregating in F2s) might also be expected if analogous studies were performed with TxM or TxN pedigrees. Again, given the inheritance of resistance from non-native species no susceptibility would be expected in hybrid pedigrees that did not include *P*. *trichocarpa*. These would include the following: DxN, DxM, or NxM. There is also likely some incomplete resistance in *P*. *trichocarpa*; a genome-wide association study may help to clarify resistance to the mite in this regard.

## Conclusion

Resistance to *S*. *mesophyllincola* was confirmed to be inherited from the non-native, D parent of interspecific, TxD hybrids, and thus an exaptation. Eastern North American *P*. *deltoides* is not native to the PNW where the mite is endemic. Secondly, of the seven types of arthropod feeding damage in the DeWoody study (i.e., chewer, skeletonizer, leaf miner, gall damage, leaf rollers, and sap-feeding arthropods on the leaf or stem), it is striking that the LG I QTL for resistance to *S*. *mesophyllincola*, a sucking arthropod, did prove to be the QTL for sap-feeding arthropods (i.e., an aphid, another sucking arthropod) in the UK. This particular QTL, associated as it was with multiple leaf developmental traits, also validated some of our ideas about putative mechanisms (i.e., veinal delimitation of bronzed areas, architecture of spongy mesophyll, leaf phenology, etc.) that might be responsible for resistance to *S*. *mesophyllincola*. Finally, it is interesting that when the categorical data from the full scale (13 categories from 0 to 100) were collapsed to just resistance (< or equal to 5) or susceptibility (> 5) we detected just a single Mendelian gene. Yet, when the full-scale scores were used we found three significant QTLs. We interpret this discrepancy as the product of greater resolution with full-scale scores and QTL analysis.

## Supporting information

S1 Table Phenotypic data for mite bronzing in the *Populus tricharpa* x *Populus deltoides* pedigrees. (XLSX)
